# Improving the adherence to COVID-19 preventive measures in the community: Evidence brief for policy

**DOI:** 10.3389/fpubh.2022.894958

**Published:** 2022-08-01

**Authors:** Izabela Fulone, Jorge Otavio Maia Barreto, Silvio Barberato-Filho, Cristiane de Cássia Bergamaschi, Luciane Cruz Lopes

**Affiliations:** ^1^Graduate Course in Pharmaceutical Sciences, University of Sorocaba, Sorocaba, Brazil; ^2^Fiocruz School of Government, Oswaldo Cruz Foundation, Brasília, Brazil

**Keywords:** COVID-19, health policy, pandemic, evidence-informed policy, knowledge translation

## Abstract

**Objectives:**

To identify evidence-based strategies to improve adherence to the preventive measures against the coronavirus disease (COVID-19) at the community level.

**Method:**

This is an evidence brief for policy, combining research evidence specific to contextual knowledge from stakeholders. A systematic search was performed in 18 electronic databases, gray literature, and a handle search, including only secondary and tertiary studies that focused on the adherence of the general population to COVID-19 preventive measures in the community. Two reviewers, independently, performed the study selection, data extraction, and assessment of the quality of the studies. Relevant evidence has been synthesized to draft evidence-based strategies to improve adherence. These strategies were circulated for external endorsement by stakeholders and final refinement. Endorsement rates >80%, 60–80% and <60% were considered high, moderate, and low respectively.

**Results:**

Eleven studies, with varying methodological qualities were included: high (*n* = 3), moderate (*n* = 3), low (*n* = 1), and critically low (*n* = 4). Three evidence based strategies were identified: i. Risk communication; ii. Health education to the general public, and iii. Financial support and access to essential supplies and services. The rates of endorsement were: 83% for risk communication, 83% for health education, and 92% for financial support and access to essential supplies and services. The evidence showed that an increase in knowledge, transparent communication, and public awareness about the risks of COVID-19 and the benefits of adopting preventive measures results in changes in people's attitudes and behavior, which can increase adherence. In addition, the guarantee of support and assistance provides conditions for people to adopt and sustain such measures.

**Conclusions:**

These strategies can guide future actions and the formulation of public policies to improve adherence to preventive measures in the community during the current COVID-19 pandemic and other epidemics.

## Introduction

In 2020, the coronavirus disease 2019 (COVID-19) quickly spread around the world, causing a global health and economic crises, unprecedented and, uncertain prospects for the period post-pandemic (1, 2).

COVID-19 is an infectious disease caused by the a novel coronavirus, severe acute respiratory syndrome-coronavirus-2 (SARS-CoV-2), that was first identified in December 2019 in China (2). With its rapid spread globally, the World Health Organization (WHO) declared the COVID-19 pandemic as an emergence of global importance. Since then, society, scientists, decisionmakers, and health systems have been challenged (3).

So far, there is no prophylactic drug treatment. As such, the disease is mostly controlled through non-pharmacological community measures and vaccination (4, 5). Vaccinations started in December 2020 in the United Kingdom, and the vaccination coverage rates vary considerably between countries. While some countries have achieved high vaccination coverage, others still lag behind. Furthermore, the emergence of several variants of the novel coronavirus, which worries the global scenery, highlights the importance of adopting the preventive measures at the community level, as these are the most effective and accessible measures currently (6, 7). The main community measures for the prevention of COVID-19 implemented in most countries include social distancing, quarantine, hand hygiene, and the use of facemasks (7, 8).

The combination of these non-pharmacological interventions aims to delay/decrease the spread of the virus and avoid the overburdening of health systems (9). Despite being simple, cheap, and effective, these measures have not achieved homogeneous adherence in the communities (10, 11). Adherence varied among the studies and mainly between the types of measures adopted (12). Furthermore, these measures were implemented differently in the countries (7) and efforts have been made to increase and sustain their acceptability, adherence, and public awareness.

The implementation and adherence to these measures are particularly more difficult in low and middle-income countries, especially in vulnerable populations, as observed in the previous epidemics (13). The poorest population, including those dependent on public transport, informal workers, homeless, people living in slums or crowded houses without adequate ventilation or without basic sanitation, are at a high risk of being infected and affected by serious crisis economic crisis (14).

In this context, it is crucial to contain the pandemic by improving the population's adherence to effective community measures for the prevention and control of COVID-19. However, the results and impact depend on collective behavior and interventions from government agencies. This study identified the best available evidence and described strategies to improve adherence to COVID-19 preventive measures in the community.

## Methods

**Figure 1:**
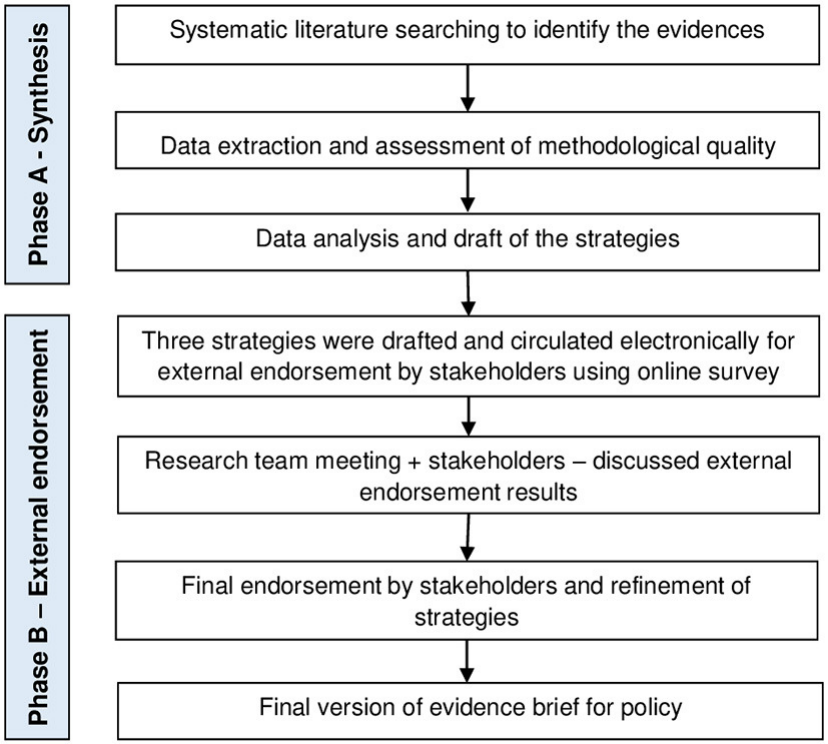
Phases to produce the evidence synthesis and its external endorsement.

We conducted an evidence brief for policy (study that package research evidence to inform deliberations among policymakers and stakeholders) (15) combining two phases: (A) synthesizing the evidence from systematic literature searching around effective strategies (interventions) to improve the adherence to COVID-19 preventive measures in the community and (B) external endorsement and evidence brief final refinement by stakeholders, Figure 1.

### Phase A: Searching and identifying the literature, data extraction and assessment of methodological quality

#### Elegibility criteria for studies

Studies were selected based on the following inclusion criteria:

#### Type of studies

We included systematic reviews (SR), rapid reviews (RR), overviews, evidence brief, clinical practice guidelines and policy guidelines. Guidelines were only considered if GRADE Evidence to Decision (EtD) was used.

#### Type of participants

Studies involving the general population exposed to COVID-19 and other severe acute respiratory syndromes (SARS).

#### Type of interventions

Any type of strategy to promote or improve adherence to community measures, such as handwashing, quarantine, social distancing, and use of facemasks for the prevention and control of COVID-19 and other SARS.

Studies that only suggested interventions to increase adherence but did not measure these were excluded.

#### Type of comparisons

There were no restrictions on the types of comparisons.

#### Type of outcome measures

Primary outcomes: number/proportion of people who adhere to community measures for the prevention and control of COVID-19 and other SARS; reduction of incident cases, hospitalizations, and mortality.

Secondary outcomes: cognitive or behavioral changes; changes in the knowledge, awareness, attitudes, acceptability, and behavior; factors associated with adherence or not adherence; knowledge/understanding of concepts or skills relevant to the critical appraisal of health claims.

### Information sources and search strategy

A systematic search was conducted in the following databases: MEDLINE, LILACS, The Cochrane Library, Health System Evidence, Health Evidence, EMBASE, CINAHL, Global Index Medicus, Epistemonikos, International Initiative for Impact Evaluation (3ie), Campbell Collaboration, Clinical Trial Registry, WHO ICTRP—International Clinical Trials Registry Platform (ICTRP) and GIN Guidelines International Network. It was also searched in some specific database for COVID-19: Coronavirus (COVID-19) Special Collections from Cochrane, COVID19 Study register from Cochrane, COVID-END from McMaster and COVID-19 Evidence database from Epistemonikos.

In addition, reference lists, gray literature and handle searches were also performed using a search strategy developed by two specialists. The supplement (Supplementary Data Sheet 1) shows the search strategy for MEDLINE which was adapted for each of the other databases. Electronic searches were conducted between January 28 to February 06, 2021.

#### Screening and selection of studies

A pilot exercise was conducted using 50 abstracts for the entire screening team to calibrate and test the review form. Subsequently, titles and abstracts were screened independently according to the selection criteria by pairs of reviewers (CB, SBF, JOMB, TB). Disagreements regarding the eligibility of studies were resolved by discussion, and a third reviewer (LCL) was consulted when necessary.

The full texts of the potentially relevant papers were retrieved for examination. The inclusion criteria were then independently applied to the full-text version of the papers by the same pairs of reviewers. Conflicts were resolved by discussion, and a third reviewer (LCL) was consulted when necessary.

#### Data extraction

The following data were extracted from the included studies: study objectives, designs, number of studies included, number and type of participants, intervention/strategy, main findings, country of study, and date of the last search. Data extraction was performed independently by the same pairs of reviewers and checked by another reviewer. Disagreements were resolved through discussion and consensus.

#### Assessment of methodological quality

The qualities of SRs were independently assessed by a pair of reviewers, using the A MeaSurement Tool to Assess systematic Reviews (AMSTAR 2) (16). The quality of RR was assessed by pair of reviewers, independently, using an adaptation of the Cochrane checklist for rapid reviews (17). See all items assessed in Supplementary Table S1.

To assess the quality of clinical practice guidelines or policy recommendations, three reviewers used the AGREE checklist (18). The AGREE II analyses of 23 items divided into 6 quality domains and two global classification items, in which the raters used a scale from 1 to 7 points (1- corresponds to “strongly disagree” and 7 “strongly agree”). The quality score was individually assigned by the reviewers and the total percentage of each domain was obtained from the following calculation: [(obtained score—minimum possible score) divided by (maximum possible score—minimum possible score)]. Scores >80%, 60–80%, and <60% were considered to be of high, sufficient and low quality, respectively. The guidelines' overall rating and recommendation were independently determined by each rater and a consensus was reached.

#### Data analysis and drafting the strategies

After extracting data, pairs of reviewers independently categorized the strategies using the taxonomy from Health Systems Evidence (19). It consists in taxonomy of governance, financial and delivery arrangements and implementation strategies within health systems^20^. We chose the topic “implementation strategies.”

Conflicts and disagreements during this process were resolved through discussion and consensus.

Data from the included studies were synthesized using tables and a narrative summary.

### Phase B—External endorsement—Incorporating knowledge from stakeholders

Key people from the health departments and committees dealing with COVID-19 from Brazil and civil society with contextual knowledge were identified. Among 29 people who were invited, 20 agreed to participate in Phase B.

We circulated the recommendations electronically to this same group of 20 stakeholders (including policymakers, frontline health professionals, researchers and civil society organization representatives) for external endorsement using an online survey.

We asked stakeholders whether they fully endorsed, did not endorse, or had no opinion about recommendations. Participants were also invited to provide comments. We considered endorsement rates of >80% as high, 60%−80% as moderate, and <60% as low levels of endorsement. The results were discussed during an online meeting with the research team and stakeholders, and the results were incorporated into the final version of the evidence brief and refinement of the strategies.

### Patient and public involvement

We had stakeholders (policy-makers, health professionals, researchers, and civil society organization representatives involved in the phase B of this project.

## Results

**Figure 2:**
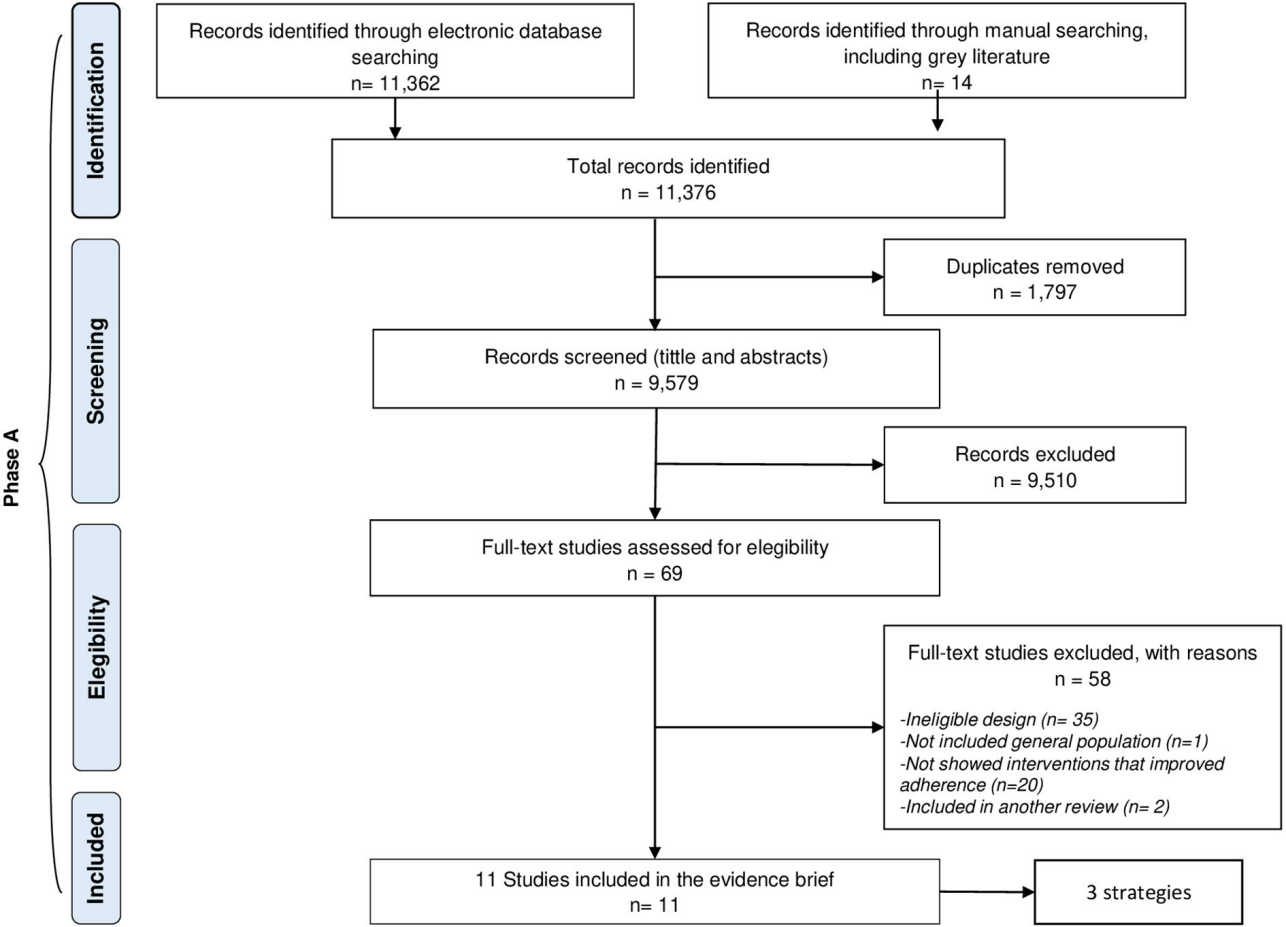
Selection of studies.

Of the 11.376 identified studies, eleven studies met the inclusion criteria, Figure 2. In the full text stage, fifty-eight studies were excluded, and their reasons are shown in the Supplementary Material (Supplementary Table S2).

Of the 11 studies, 6 were RR (11, 20–24), 4 SR (25–28), and one guideline for policy (29). From these studies, we identified 3 strategies and categorized them as followed: i. Risk communication (6 studies included it); ii. Health education to the general public (4 studies included it), iii. Financial support and access to essential supplies and services (2 studies included it), Table 1. Characteristics of included studies, see Supplementary Table S3.

**Table 1:** Characteristics of the studies included for identifying policy strategies.

**Author, year**	**Number/type of studies included in the review**	**Number of participants**	**Main outcomes**	**Which preventive** **measure does study refer to?**	**Taxonomic classification of policy strategy^&^**
Winograd et al. (24)	31 studies: *14 RCTs, 17 nonRCTs*	40,183^*^	Cognitive or behavioral outcomes	Multiples^#^	Risk communication
Webster et al. (21)	14 studies: *6 qualitative studies, 8 quantitative studies*	52,029	Factors associated with adherence or non-adherence	Social distancing	Support/access
Li et al. (20)	24 *cross-sectional studies*	35,967	Knowledge, attitude, practice or awareness	Multiples^#^	Health education
Mills et al. (22)	89 *cross-sectional studies*	Not reported	Factors contributing to facemask use	Facemasks use	Risk communication
NCCMT (11)	17 studies: *9 secondary studies, 5 primary studies, 3 guidances*	Not reported	Change in knowledge, attitudes and behavior	Multiples^#^	Risk communication
Ryan et al. (23)	31 studies: *16 primary studies, 1 RR, 1 review of guideline, 8 SR, 3 guidelines, 2 reviews/meetings analyses*	Not reported	Increased acceptability and adherence to social distance	Social distancing/ quarantine	Risk communication Support/access
Cusack et al. (28)	24 studies: *14RCTs, 10 non-RCTs*	16,530^*^	knowledge or understanding of concepts/skills relevant to evaluating the effects of, or claims about, health interventions	Multiples^#^	Health education
WHO (29)	13 studies: *12 SR, 1 RR*	Not reported	Adoption of preventive behavior	Multiples^#^	Risk communication
Solhi et al. (27)	16 studies: *4 before-and-after studies, 12 intervention-control studies*	10,960	Prevention or reduction of the incidence of infectious diseases	Multiples^#^	Health education
Nordheim et al. (26)	8 studies: *1 RCT, 7 non-RCTs*	1,148^*^	Critical appraisal abilities for health claims	Multiples^#^	Health education
FitzpatrickLewis et al. (25)	24 studies: *21 quantitative studies, 3 qualitative studies*	3,546	Awareness, knowledge, attitude or behavioral change	Multiples^#^	Risk communication

* One of the studies did not report the number of participants.

# Preventive measures defined in this evidence brief and others types;

& the classification of strategies was done according to the Health Systems Evidence Taxonomy; RCT, randomized controlled trial; non-RCT, non-randomized controlled trial; RR: rapid review; SR, systematic review.

The methodological quality of studies was varied. Of the 4 SR, 2 were moderate-quality (26, 28), 1 low-quality (25), and 1 critically low quality (27). Of the 6 RR, 2 were high-quality (11, 23), 1 moderate quality (20), and 3 critically low quality (21, 22, 24), Table 2.

**Table 2:** Methodological quality of systematic reviews and rapid review according to AMSTAR 2 and adapted Cochrane checklist respectively.

**Author, year**	**Q1**	**Q2**	**Q3**	**Q4**	**Q5**	**Q6**	**Q7**	**Q8**	**Q9**	**Q10**	**Q11**	**Q12**	**Q13**	**Q14**	**Q15**	**Q16**	**Ranking of Quality**
**Strategy 1: risk communication**																	
***Rapid reviews***																	
Winograd et al. (24)	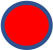	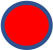	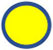	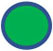	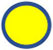	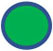	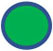	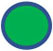	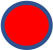	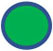	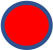	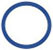	-	-	-	-	Critically low
NCCMT (11)	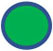	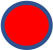	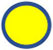	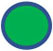	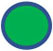	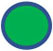	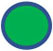	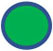	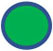	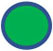	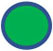	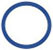	-	-	-	-	High
Ryan et al. (23)	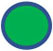	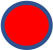	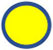	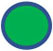	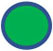	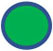	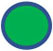	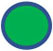	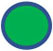	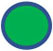	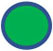	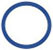	-	-	-	-	High
Mills et al. (22)	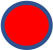	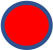	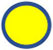	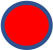	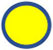	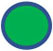	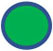	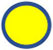	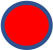	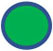	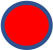	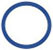	-	-	-	-	Critically low
***Systematic review***																	
Fitzpatrick-Lewis et al. (25)	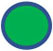	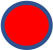	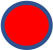	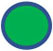	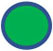	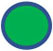	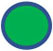	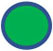	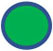	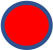	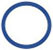	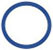	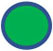	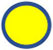	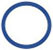	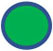	Low
**Strategy 2: health education to the general public**																	
***Rapid reviews***																	
Li et al. (20)	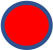	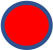	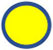	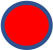	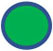	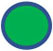	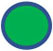	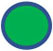	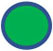	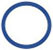	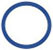	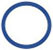	-	-	-	-	Moderate
***Systematic reviews***																	
Cusack et al. (28)	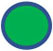	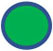	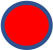	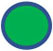	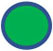	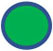	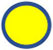	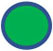	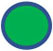	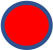	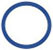	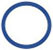	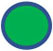	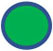	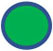	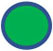	Moderate
Solhi et al. (27)	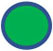	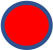	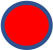	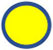	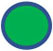	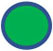	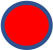	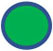	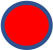	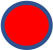	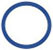	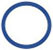	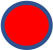	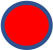	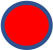	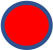	Critically low
Nordheim et al. (26)	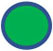	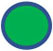	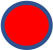	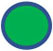	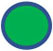	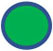	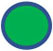	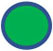	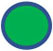	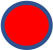	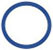	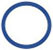	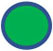	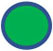	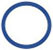	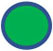	Moderate
**Strategy 3: financial support and access to essential supplies and services**																	
***Rapid reviews***																	
Ryan et al. (23)	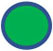	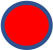	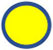	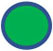	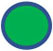	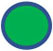	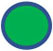	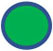	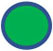	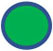	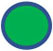	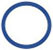	-	-	-	-	High
Webster et al. (21)	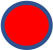	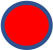	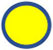	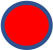	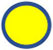	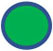	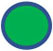	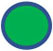	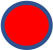	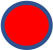	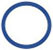	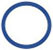	-	-	-	-	Critically low

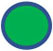
yes, 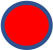
no; 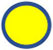
partially yes, 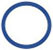 not applicable (meta-analysis not performed).

The only guideline included had high quality (Supplementary Table S4).

### Strategies identified

#### Strategy 1— Risk communication

Six studies, with varying methodological qualities qualities (three high quality, three moderate quality), were included. Risk communication is defined as the “exchange of information, advice and opinions, in real-time, among experts, community leaders or officials, and people at risk who face threats to their health and social well-being” (29). This strategy should be based on three pillars, as shown below (29):

##### Building trust

The information must be easily found in legitimate/reliable sources, and it should be clear, consistent, unified, practical, and up-to-date. It should discuss the risks (dissemination, contagion, and severity of COVID-19), the benefits, the need, the effectiveness and the rationality of adopting community measures to prevent COVID-19 (11, 29). The population must receive practical information on what they should do and for how long.

These messages must be constantly reinforced (29) and disseminated widely across different media, including traditional, social, local, and mobile media (25). It is also important to maintain proactive communication from government and official authorities and monitor public perception, uncertainties, concerns, and inconsistencies in the population (23).

##### Transparency

Communicating and recognizing uncertainties, errors, and changes in information. Negative information, such as the number of victims, should not be occulted (11, 29).

##### Community participation

Identification and involvement of people that the community trusts (trusted leaders, be it a health professional or a public health leader) in the development and dissemination of the messages (11, 29). The messages should be customized according to the target audience, the cultural context, and their understanding, involving stakeholders to ensure the flow, integrating the community into practice (23, 29). These should also be tested in advance using a small group from the community (29). The messages should reinforce social responsibility and a sense of altruism to increase the population's motivation to adhere to the preventive measures (22).

In this way, risk communication is effective as it produces cognitive changes in the perception of disease risk, which in turn encourages behavior change and increases adherence to preventive measures (24). Repeatedly providing clear information on how the virus is transmitted, the risk of contagion, the health risk, the severity of the disease (perception of risk), as well as the effectiveness and benefits at the individual and community (perception of benefit) levels of the preventive measures allowed people to understand and adopt preventive measures (11, 22, 23). Trust in science, local and/or national government institutions, and in the person delivering the message, and the sense of altruism (protecting oneself and others) are important in influencing behavior and increasing adherence (11, 22, 23).

Failures in communication can have negative effects on the acceptability and adherence to preventive measures (22, 23). Conflicting or confusing information taints the credibility and reliability of the information, which can cause “information fatigue” (22). The public is skeptical, and messages are considered alarmists (23). Delays in the transmission of messages by officials and agencies, government failure, and misinterpretation reduce public trust and discourage the population from continuing to adhere to preventive measures (22, 23).

#### Strategy 2—Health education to the general public

Four studies with varying methodological qualities (three moderate quality and one critically low) were included.

Health education refers to any type of combination of learning experiences designed to help individuals and communities improve their health, increase their knowledge, and/or influence their attitudes (30). It is a permanent pedagogical process of building knowledge. However, it is not only limited to the dissemination of information related to health (30) as it must involve the promotion of motivation, skills, trust, and necessary autonomy to act and improve health and adopt healthy and preventive practices.

This strategy plays a key role in the prevention and control of emerging infectious diseases (27). It should be broad, consistent, released as early as possible. Furthermore, it should be focused on public awareness of COVID-19 (risk of transmission, symptoms, and measures to reduce the spread of virus), prevention (the benefits and effectiveness of adopting adequate preventive measures), adoption of adequate face mask use (how to use and wash them), hand hygiene (wash your hands well with soap and water or use alcohol gel). Lastly, it should utilize of reliable information and sources related to the pandemic (20, 27).

Health education increases knowledge and public awareness, which improves attitudes, practices, and behaviors during the pandemic (20). It also contributes to maintaining optimistic attitudes and reducing the level of anxiety, tension, fear, and depression. As such, it may be more effective in the most vulnerable groups or in those who commonly adopt risk behaviors, such as young people (20). The most recommended types of educational interventions in this current pandemic involve raising awareness through national media campaigns and web-based educational programs (20, 27). This strategy is also effective in improving the understanding of key concepts related to health and skills in critically assessing health issues, including the general public (28) and teenagers (26).

#### Strategy 3—Financial support and access to essential supplies and services

Two studies with varying methodological qualities (one high-quality and one critically low quality).

Several practical elements need to support or sustain behavior change and the population's response to certain preventive measures, especially quarantine or social distancing. The population may have the desire, motivation, and knowledge to adhere to preventive measures, but if there are no means to do so, they will not adhere (31).

It is essential to offer certain types of support/assistance to the population, especially those most affected by the pandemic, and ensure that they know clearly which are available and how they can be accessed (23). This should include: financial support and access to basic supplies, such as food and medicines; facilitation of access to usual and specialized medical services; maintenance of direct lines for support and communication with a team of healthcare professionals, including online services or by telephone; and implementation of measures to compensate for financial losses or job loss (21, 23).

Despite the economic and social impact, ensuring of financial support and access to essential supplies increases adherence to preventive measures and mitigates long-term effects on the physical and mental health of the population. Hard-to-access support services cause stress and non-adherence (23), and the fear of losing one's job and family income were the main nonadherence factors (21).

#### External endorsement results

Twenty stakeholders participated and completed the online endorsement survey, of which 2 (10%) were policy-makers, 2 (10%) frontline health professionals, 13 (65%) were researchers (professionals who work in research institutes focused on decision making), and 3 (15%) were civil society organization representatives. The rates of total endorsement for the recommendations were: 83% for risk communication, 83% for health education, and 92% for financial support and access to essential supplies and services.

Emphasis was placed on the importance of customizing risk messages according to the target audience and on empowering the primary health care team in the elaborating and disseminating these messages. Participants tended to endorse the health education, but they highlighted that it would be the most challenging recommendation during a pandemic. Furthermore, e-learning, one of the most commonly used forms, could have difficulties impacting a specific part of the population. Support was considered the most important because it enables the adoption of preventive measures during the pandemic.

## Discussion

This evidence brief presented strategies for improving the adherence to COVID-19 prevention measures in the community using complex knowledge synthesis of evidence from literature and contextual expert knowledge from stakeholders. The available evidence from 11 studies identified three strategies that may be useful in dealing with non-adherence, which was highly endorsed by stakeholders.

The time required to produce evidence is not always the same for decision making. Sometimes, robust evidence obtained from studies of high methodological quality may not be available when decision-makers need it, as in this case. The strategies were derived from some low-quality studies with varied evidence levels. As such, we included information on uncertainties and gaps.

There were more studies for strategy 1 than for other strategies; however, the quality was quite varied. Different approaches to risk communication were promising, but it was not possible to determine the best approach (24, 25). Evidence has showed that interventions targeting a specific population were more effective than the ones that do not (24). Another point highlighted in all the studies included was the importance of community involvement in the elaboration and transmission of risk messages, which increases acceptability, trust, and adherence to preventive measures (11, 23, 29).

Despite the relevance of the role of the strategy 2 in emerging diseases, the format of the educational process became more complicated in a pandemic scenario. The studies only suggested providing health education by media campaigns, telephone, or web-based programs (20, 27). In general, the format was variable, and little information about the educating agent was provided in the studies. The long-term effects of health education are still unknown because the studies assessed immediately after or shortly (after 28 weeks) (27). The lack of health education and low health literacy (32) poses a big risk in the COVID-19 pandemic due to the proliferation of false information (misinfodemic). When misinformation or false news is disseminated repeatedly, the marginal impact of true information on the population is limited (22), which might influence people's health decisions and encourage unhealthy behaviors. As health information and misinformation have become more abundant in recent years, through mass media or the internet, it has become increasingly crucial to have general knowledge and skills to assess whether claims about health interventions are trustworthy (28). Studies have shown that educational interventions can improve knowledge and skills in the critical appraisal of health claims at least in the short term (26, 28).

Strategy 3 shows the essential elements to support and sustain preventive measures, especially quarantine. Financial support and access to essential supplies (food and medicines) and services (usual and specialized medical services) result in significant economic and social impacts, but robust economic assessments are needed (23). Some other positive support/access strategies were cited in some studies, but they did not assess their effectiveness in improving adherence, such as the provision of stations for handwashing, water, soap, or alcohol-gel to increase adherence to hand hygiene, whether in a public environment or domicile (13), distribution of masks in order to increase adherence to the use of masks (13); provision of quarantine or isolation centers (13); and control/reduction in the number of passengers on public transport (33).

The impact of strategy 3 may be even greater in low-income countries groups, or places that are potentially at a disadvantage. These include populations without clean water and those unable to buy masks, soap, or alcohol, or to keep their distance in crowded houses or slums.

The diverse group of stakeholders engaged in the endorsement process ensured the review, assessment of the feasibility, and refinement of the strategies in real practice. This phase was important as it weighed the research evidence with the knowledge, values, insights, and experiences of stakeholders. The approach of including stakeholders in the process has grown in the past several years and is critical for implementing promising interventions and improving the health of communities (34).

Bringing together evidence producers and users contributes to the dissemination and application of global and local evidence in real-world settings, reducing the gap between research and practice.

Although it was not the focus of our research and was not discussed among stakeholders, it is important to note that both strategies 1 and 2 could also help in the vaccination adherence to COVID-19. Vaccine hesitancy is another major challenge that countries are facing probably due negative and unclear information spread by media, lack of education and health literacy (35, 36).

Observational studies conducted in some countries identified that high adherence to COVID-19 preventive measures was associated with willingness to vaccinate against the disease (36, 37). The known major predictors that affect the adherence to preventive measures are similar to vaccination adherence, such as age, socioeconomic status, education level, health literacy level, trust in their government and in their healthcare system (36, 37). Women, older age, with chronic disease, higher education levels, higher health literacy, life satisfaction were associated positively to adherence to preventive measures and to vaccination (37).

Risk communication and health education could contribute to address positive and true information about the COVID-19 vaccination, to avoid the spread of misinformation, reduce disbeliefs, hesitancy and resistance, increase the confidence within the population about the benefits of vaccination and the percentage of vaccine definite people (35, 36).

Finally, our findings could be used by practitioners and policy-makers working in the field of prevention and control of COVID-19 to improve the adherence to COVID-19 preventive measures and the willingness of the community to vaccine and combat COVID-19.

### Strengths and limitations

To our knowledge, this was the first study to address this objective and involved relevant stakeholders for the endorsement of effective strategies.

While the three strategies are well outlined, it is important to mention that social influences could be a key motivation for some people's adherence to COVID-19 preventive measures. For instance, people have more motivation to adherence when their close social circle did (38).

The variability in the quality of the included studies may cause some uncertainties and limit the confidence in the findings. Further studies should be conducted to assess the effectiveness of interventions to improve adherence to preventive measures in the community for future epidemics. Furthermore, rigorous methods in order to provide high-quality evidence.

The majority of the studies also focused on high-income countries. This limits their application in low-income countries, which face different challenges, mainly in relation to its implementation. Additionally, if those implementing the strategy do not take into account and nurture the local culture, the project is doomed. Therefore, more studies need to be conducted in low- and middle-income countries.

## Conclusion

Based on the best available evidence, this evidence brief identified three strategies for improving adherence to preventive measures against COVID-19 in the community, which may guide future actions and policymaking during this pandemic or future epidemics. In addition, two of these strategies could contribute to improve the vaccination adherence and reduce the hesitancy and resistance in the community.

The intention is not to recommend specific strategies but to inform policymakers and stakeholders and contribute to assertive decision-making in public health, according to the needs, financial resources, feasibility, local reality, and the engagement of the main actors. This evidence brief provides relevant information for planners and policymakers to choose the most effective strategy.

## Data availability statement

The original contributions presented in the study are included in the article/supplementary material, further inquiries can be directed to the corresponding author.

## Ethics statement

The studies involving human participants were reviewed and approved by the Research Ethics Committee of the University of Sorocaba, on 15 October 2020, (CAAE: 38351620.9.0000.5500) according to Resolution CNS 466/12 of the National Health Council. The patients/participants provided their written informed consent to participate in this study.

## Author contributions

IF and LL led the conceptual design of the original study, acquisition of funding, drafted the manuscript and conducted the methodological quality assessment. JB, SB-F, CB, and IF selected tittles and abstracts and extracted data from included studies. All authors read and approved the final manuscript.

## Funding

National Council for Scientific and Technological Development—CNPq, Brazil, grants n°401924/2020-3 and Ministry of Health (MOH).

## Conflict of interest

The authors declare that the research was conducted in the absence of any commercial or financial relationships that could be construed as a potential conflict of interest.

## Publisher's note

All claims expressed in this article are solely those of the authors and do not necessarily represent those of their affiliated organizations, or those of the publisher, the editors and the reviewers. Any product that may be evaluated in this article, or claim that may be made by its manufacturer, is not guaranteed or endorsed by the publisher.
