# Supplementary material for: Improving the adherence to COVID-19 preventive measures in the community: Evidence brief for policy
Source: Front Public Health. 2022 Aug 1;10:894958. doi: 10.3389/fpubh.2022.894958 (PMC9376604; doi:10.3389/fpubh.2022.894958)
Supplement: Supplementary file 1 [file Data_Sheet_1.DOCX]

**SUPPLEMENTARY DATA SHEET 1 – SEARCH STRATEGY**

**Database: MEDLINE, Ovid – <1946 to January, 28, 2021>**

1. exp Coronavirus/

2. exp Coronavirus Infections/

3. SARS Virus/

4. Middle East Respiratory Syndrome Coronavirus/

5. Pneumonia, Viral/

6. Influenza, Human/

7. Pandemics/

8. Epidemics/

9. exp Communicable Diseases/

10. Disease Outbreaks/

11. Space-Time Clustering/

12. (coronavir* or coronovirus* or betacoronavir* or beta-coronavirus or beta-coronaviruses or corona virus or virus corona or corono virus or virus corono or hcov* or covid* or 2019-ncov or cv19* or cv-19 or cv 19 or n-cov or ncov* or (wuhan* and (virus or viruses or viral)) or sars* or sari or severe acute respiratory syndrome or mers* or middle east respiratory syndrome or middle-east respiratory syndrome or 2019-ncov-related or cv-19-related or n-cov-related or pandemic* or epidemic*).ti,ab,kf.

13 1 or 2 or 3 or 4 or 5 or 6 or 7 or 8 or 9 or 10 or 11 or 12

14. Social Isolation/

15. Quarantine/

16. Hand Disinfection/

17. Hand Hygiene/

18. Personal Protective Equipment/

19. Masks/

20. (isolate* or distancing* or quarantin* or handwash* or (hand? adj2 (wash* or clean* or disinfect* or hygien*)) or hand saniti* or personal protect* or facemask* or face mask* or protective measure*).ti,ab,kf.

21. 14 or 15 or 16 or 17 or 18 or 19 or 20

22. Health Promotion/

23. Patient Education as Topic/

24. Health Knowledge, Attitudes, Practice/

25. Health Behavior/

26. (Treatment Adherence and Compliance).mp. [mp=title, abstract, original title, name of substance word, subject heading word, floating sub-heading word, keyword heading word, organism supplementary concept word, protocol supplementary concept word, rare disease supplementary concept word, unique identifier, synonyms]

27. "Patient Acceptance of Health Care"/

28. Patient Compliance.mp. or Patient Compliance/

29. Guideline Adherence/

30. Cooperative Behavior/

31. Preventive medicine.mp. or Preventive Medicine/

32. Preventive health services.mp. or Preventive Health Services/

33. Public health.mp. or Public Health/

34. Attitude to Health/

35. 22 or 23 or 24 or 25 or 26 or 27 or 28 or 29 or 30 or 31 or 32 or 33 or 34

36. 13 and 21 and 35
