# Supplementary material for: Improving the adherence to COVID-19 preventive measures in the community: Evidence brief for policy
Source: Front Public Health. 2022 Aug 1;10:894958. doi: 10.3389/fpubh.2022.894958 (PMC9376604; doi:10.3389/fpubh.2022.894958)
Supplement: Supplementary file 2 [file Table_1.DOCX]

**SUPPLEMENTARY TABLE S1 - ASSESSMENT OF THE METHODOLOGICAL QUALITY OF INCLUDED STUDIES.**

**Table S1: Items assessed in systematic reviews and rapid reviews.**

|  | **Items assessed in systematic review**  **(AMSTAR 2)** | **Items assessed in rapid reviews**  **(Adapted Cochrane checklist)** |
| --- | --- | --- |
| **Q1**  Yes  Partially yes  No  Meta-analysis was not performed | **Q1 -** Did the research questions and inclusion criteria for the review include the components of PICO (population, intervention, control group, and outcome)? | **Q1**- Was there stakeholder involvement to refine the research question, PICO, eligibility criteria, and the outcomes of interest? |
| **Q2** | **Q2 (critical domain) –** Did the report of the review contain an explicit statement that the review methods were established prior? | **Q2**- Did a priori protocol developed containing the research question, PICO, and inclusion and exclusion criteria? |
| **Q3** | **Q3** - Did the review authors explain their selection of the study designs for inclusion in the review? | **Q3**- Was the research question (PICO) clearly defined considering the most important outcomes for the health decision? |
| **Q4** | **Q4 (critical domain) -** Did the review authors use a comprehensive literature search strategy? | **Q4**- Did it emphasize high-quality study designs or considered a stepwise approach to including study designs? |
| **Q5** | **Q5 -** Did the review authors perform study selection in duplicate? | **Q5 (critical domain)** – Was the search strategy done on at least three databases? If there was a restriction on the publication date, was there a methodological or clinical justification? Did it involve a librarian in developing the search strategy? |
| **Q6** | **Q6** - Did the review authors perform data extraction in duplicate? | **Q6 (critical domain)** - Was the selection of studies (titles, abstracts and full texts) made in duplicate? Or was the selection done in duplicate in at least 20% of the studies and conflict resolution? Or did a single reviewer select the studies and a second reviewer selected the excluded studies, with conflict resolution? |
| **Q7** | **Q7** **(critical domain)** - Did the review authors provide a list of excluded studies and justify the exclusions? | **Q7 (critical domain)** - Was the data extraction done in duplicate? Or was the extraction done by a single reviewer and a second reviewer checked the correctness and completeness of e extracted data? |
| **Q8** | **Q8** - Did the review authors describe the included studies in adequate detail? | **Q8 (critical domain**)- Was a valid tool used to assess the risk of bias of the included studies? |
| **Q9** | **Q9 (critical domain) -** Did the review authors use a satisfactory technique for assessing the risk of bias in individual studies that were included in the review? | **Q9 (critical domain)** - Was the risk of bias assessment done in duplicate? Or was it done by a single reviewer and a second reviewer did the full verification of the judgments? |
| **Q10** | **Q10** - Did the review authors report on the sources of funding for the studies included in the review? | **Q10 (critical domain**) - Was an adequate tool used to grade the certainty of evidence? |
| **Q11** | **Q11** **(critical domain) -** If meta-analysis was performed, did the review authors use appropriate methods for statistical combination of results? | **Q11 (critical domain**)- Was the certainty grade of the evidence done in duplicate? Or was the certainty of the evidence rated by a single reviewer and a second reviewer did the full verification of the judgments? |
| **Q12** | **Q12** - If meta-analysis was performed, did the review authors assess the potential impact of RoB in individual studies on the results of the meta-analysis or other evidence synthesis? | **Q12**- If it was possible to perform a meta-analysis, were appropriate statistical methods used to combine the results? |
| **Q13** | **Q13 (critical domain) -** Did the review authors account for RoB in primary studies when interpreting/discussing the results of the review? | - |
| **Q14** | **Q14** - Did the review authors provide a satisfactory explanation for, and discussion of, any heterogeneity observed in the results of the review? | - |
| **Q15** | **Q15** **(critical domain) -** If they performed quantitative synthesis did the review authors carry out an adequate investigation of publication bias and discuss its likely impact on the results of the review? | - |
| **Q16** | **Q16 -** Did the review authors report any potential sources of conflict of interest, including any funding they received for conducting the review? | - |
