# Supplementary material for: Improving the adherence to COVID-19 preventive measures in the community: Evidence brief for policy
Source: Front Public Health. 2022 Aug 1;10:894958. doi: 10.3389/fpubh.2022.894958 (PMC9376604; doi:10.3389/fpubh.2022.894958)
Supplement: Supplementary file 3 [file Table_2.DOCX]

**SUPPLEMENTARY TABLE S2 – EXCLUDED STUDIES**

**Table S2. Excluded studies after reading the full texts.**

| **Author, year** | **Tittle of study** | **Reason of exclusion** |
| --- | --- | --- |
| Aathira, 2021 | Role of government during a quarantine - A review. | Ineligible Study Design |
| Abidullahi, 2020 | Community interventions in Low—And Middle-Income Countries to inform COVID-19 control implementation decisions in Kenya: A rapid systematic review | It did not present interventions that increased adherence |
| Ahsan, 2020 | Combating COVID-19 in Bangladesh: ideal duration of mandatory quarantine period and policy implications | Ineligible Study Design |
| Aimone, 2010 | The 1918 Influenza Epidemic in New York City: A Review of the Public Health Response | Ineligible Study Design |
| Alerdot, 2007 | Non-pharmaceutical public health interventions for pandemic influenza: an evaluation of the evidence base | Ineligible Study Design |
| Alisson, 2008 | Effect of Hand Hygiene on Infectious Disease Risk in the Community Setting: A Meta-Analysis | It did not present interventions that increased adherence |
| Amaechi, 2020 | Social approaches to COVID-19 pandemic response: effectiveness and practicality in sub-Saharan Africa | Ineligible Study Design |
| Bassem, 2020 | The importance of health promotion in the prevention of COVID-19 | Ineligible Study Design |
| Bassi, 2020 | An overview of mobile applications (apps) to support the coronavirus disease 2019 response in India | Ineligible Study Design |
| Bekele, 2020 | Patterns and associated factors of COVID-19 knowledge, attitude, and practice among general population and health care workers: A systematic review | It did not present interventions that increased adherence |
| Berhe, 2020 | Global epidemiology, pathogenesis, immune response, diagnosis, treatment, economic and psychological impact, challenges, and future prevention of COVID-19: A Scoping review | Ineligible Study Design |
| Bish, 2009 | Demographic and attitudinal determinants of protective behaviours during a pandemic: A review | Ineligible Study Design |
| National Academies of Sciences, Engineering, and Medicine, 2020 | Encouraging Adoption of Protective Behaviors to Mitigate the Spread of COVID-19: Strategies for Behavior Change | Ineligible Study Design |
| Bonine, 2020 | Strategies for Promoting Hand Hygiene Compliance on Inpatient Psychiatric Units. | Ineligible Study Design |
| Braunack-Mayer, 2010 | Including the public in pandemic planning: a deliberative approach | Ineligible Study Design |
| Bults, 2015 | Perceptions and Behavioral Responses of the General Public During the 2009 Influenza A (H1N1) Pandemic: A Systematic Review | It did not present interventions that increased adherence |
| Campbell, 2020 | A systematic analysis of online public engagement with 10 videos on major global health topics involving 229459 global online viewers | Ineligible Study Design |
| Chisale, 2020 | Community-based interventions for preventing COVID-19 transmission in low- and middle-income countries: A systematic review | It did not present interventions that increased adherence |
| Chu, 2020 | Social consequences of mass quarantine during epidemics: a systematic review with implications for the COVID-19 response | It did not present interventions that increased adherence |
| Compton, 2018 | Compliance with hand washing guidelines among visitors from the community to acute care settings: A Scoping Review | Ineligible Study Design |
| Cookson, 2009 | Comparison of national and subnational guidelines for hand hygiene | It did not include the general population |
| Cugelma, 2011 | Online Interventions for Social Marketing Health Behavior Change Campaigns: A Meta-Analysis of Psychological Architectures and Adherence Factors | Included in another systematic review |
| Curtis, 2009 | Planned, motivated and habitual hygiene behaviour: an eleven-country review | Ineligible Study Design |
| Davey, 2008 | Effective, Robust Design of Community Mitigation for Pandemic Influenza: A Systematic Examination of Proposed US Guidance | Ineligible Study Design |
| Ding, | Transnational Quarantine Rhetorics: Public Mobilization in SARS and in H1N1 Flu | Ineligible Study Design |
| Finset, 2020 | Effective health communication – a key factor in fighting the COVID-19 pandemic | Ineligible Study Design |
| Frakgou 2020 | Review of trials currently testing treatment and prevention of COVID-19 | It did not present interventions that increased adherence |
| Fung, 2007 | How often do you wash your hands? A review of studies of hand-washing practices in the community during and after the SARS outbreak in 2003 | Ineligible Study Design |
| Getaneh, 2020 | Global lessons and Potential strategies in combating COVID-19 pandemic in Ethiopia: Systematic Review | It did not present interventions that increased adherence |
| Ghio, 2020 | What influences people’s responses to public health messages for managing risks and preventing infectious diseases? A rapid systematic review of the evidence and recommendations | Included in another systematic review |
| Kaim, 2020 | Impact of a Brief Educational Intervention on Knowledge, Perceived Knowledge, Perceived Safety, and Resilience of the Public During COVID-19 Crisis | Ineligible Study Design |
| Kondylakis, 2020 | COVID-19 Mobile Apps: A Systematic Review of the Literature | It did not present interventions that increased adherence |
| Kurniawat, 2020 | The Policy of Large-Scale Social Restriction (LSSR): Prevention Effort of COVID-19 and Community Compliance in Indonesia | Ineligible Study Design |
| Leppin, 2009 | Risk Perceptions Related to SARS and Avian Influenza: Theoretical Foundations of Current Empirical Research | Ineligible Study Design |
| Laranjo, 2014 | The influence of social networking sites on health behavior change: A systematic review and meta-analysis | It did not present interventions that increased adherence |
| Majid, 2020 | Knowledge, (mis-)conceptions, risk perception, and behavior change during pandemics: A scoping review of 149 studies | Ineligible Study Design |
| Megnin-Viggars, 2020 | Facilitators and barriers to engagement with contact tracing during infectious disease outbreaks: A rapid review of the evidence | It did not present interventions that increased adherence |
| Moradali, 2020 | Epidemiology, Prevention and Control Strategies of Coronavirus COVID 19 in Iran: A Systematic Review | It did not present interventions that increased adherence |
| Moran, 2016 | A Meta-Analysis of the Association between Gender and Protective Behaviors in Response to Respiratory Epidemics and Pandemics | It did not present interventions that increased adherence |
| Muhammad, 2020 | Policy Brief on Child Protection during Covid-19 Crisis in Pakistan | It did not present interventions that increased adherence |
| Patino-Lugo, 2020 | Non-pharmaceutical interventions for containment, mitigation and suppression of COVID-19 infection | It did not present interventions that increased adherence |
| Regmi, 2020 | Factors impacting social distancing measures for preventing coronavirus disease 2019 [COVID-19]: A systematic review | It did not present interventions that increased adherence |
| Rothstein, 2019 | Ensuring Compliance with Quarantine by Undocumented Immigrants and Other Vulnerable Groups: Public Health Versus Politics | Ineligible Study Design |
| Sarti, 2020 | Organization of Primary Health Care in pandemics: a rapid systematic review of the literature in times of COVID-19 | It did not present interventions that increased adherence |
| Sharma, 2019 | Scoping review of non-pharmacological interventions to control H1N1 in India | Ineligible Study Design |
| Teasdale, 2014 | Public perceptions of non-pharmaceutical interventions for reducing transmission of respiratory infection: systematic review and synthesis of qualitative studies | It did not present interventions that increased adherence |
| Olry, 2020 | El uso de las mascarillas en la protección de las infecciones respiratorias: una revisión de revisiones | Ineligible Study Design |
| Sathian, 2020 | Impact of COVID-19 on community health: A systematic review of a population of 82 million | It did not present interventions that increased adherence |
| Savoia, 2013 | Communications in Public Health Emergency Preparedness: A Systematic Review of the Literature | It did not present interventions that increased adherence |
| Seale, 2020 | Improving the impact of nonpharmaceutical interventions during COVID-19: examining the factors that influence engagement and the impact on individuals | Ineligible Study Design |
| Sim, 2014 | The use of facemasks to prevent respiratory infection: a literature review in the context of the Health Belief Model | Ineligible Study Design |
| Xu, 2020 | China’s practice to prevent and control COVID-19 in the context of large population movement | Ineligible Study Design |
| Walsh, 2021 | The use of social media as a tool for stakeholder engagement in health service design and quality improvement: A scoping review | Ineligible Study Design |
| Webb, 2010 | Using the Internet to Promote Health Behavior Change: A Systematic Review and Meta-analysis of the Impact of Theoretical Basis, Use of Behavior Change Techniques, and Mode of Delivery on Efficacy | It did not present interventions that increased adherence |
| Welch, 2018 | Interactive social media interventions for health behaviour change, health outcomes, and health equity in the adult population. | Ineligible Study Design |
| Who, 2020 | Mask use in the context of COVID-19 – Interim guidance | Ineligible Study Design |
| WHO, 2020 | Recommendation to Member States to improve hand hygiene practices widely to help prevent the transmission of the COVID-19 virus | Ineligible Study Design |
| WHO, 2020 | Risk communication and community engagement readiness and response to coronavirus disease (COVID-19 – Interim guidance | Ineligible Study Design |
