# Supplementary material for: Improving the adherence to COVID-19 preventive measures in the community: Evidence brief for policy
Source: Front Public Health. 2022 Aug 1;10:894958. doi: 10.3389/fpubh.2022.894958 (PMC9376604; doi:10.3389/fpubh.2022.894958)
Supplement: Supplementary file 4 [file Table_3.DOCX]

**SUPPLEMENTARY S3 - CHARACTERISTICS OF INCLUDED STUDIES**

**Table S3.1: Characteristics of included studies about strategy 1 – Risk communication**

| **Author, year** | **Objective of study** | **Population of study** | **Main findings ^#^** | **Proportion of studies that included a target population** | **Proportion and identification of studies carried out in low- and middle-income countries*** | **Proportion of problem-focused study** | **Date of the search** |
| --- | --- | --- | --- | --- | --- | --- | --- |
| Winograd et al., 2021 | Examine whether risk communication can change people's cognition and behavior and mitigate the spread of viral illnesses. | Population over 18 years old who have received some form of risk communication aimed at viral diseases transmitted between humans. | Risk communication produced cognitive and behavioral changes around viral diseases. There was no consistency about which was the best approach, however risk communication target to a specific target audience had better outcomes than those not target. | 8/31 | Not  reported | 5/31 | Not  reported |
| NCCMT, 2020 | Identify best practices for risk communication and strategies for mitigating risk behaviors. | General population from community setting | i. Risk communication should be clear, actionable and delivered by a trusted person, such as a trusted community leader or health care provider; ii. Confidence in the risk message and the person who deliver it is built by presenting uncertainties, errors and changes in information; iii. Stakeholder engagement helps to tailor messages appropriately to context and audience; iv. Positive messages that emphasize the collective versus the individual showed to be more effective. | 8/17 | The countries of all included studies were not reported. | 8/17  (Two of these are in progress) | Set.  2020 |
| Mills et al., 2020 | To investigate the effectiveness of different types of facemasks, public messages and behavioral adherence by the general population. | People from community who have experienced epidemics caused by SARS-CoV-1, SARS-CoV-2, MERS or H1N1. | Effective and consistent risk messages are essential to ensure adherence to mask use by the general population. For this, it is recommended that the messages are clear, simple, transparent, transmitted early by the authorities, and that they focus especially on: the transmission of the virus and the risk to health.  Conflicting recommendations, without a clear and reliable source of information, generate confusion, lack of adherence on the part of the population, who can become skeptical and start to consider them alarmist. | Not reported clearly | 0/89 | Not reported clearly | 2020 |
| Ryan et al., 2020 | Identify relevant, viable and effective approaches to promote acceptability, understanding and adherence to physical distancing measures for the prevention and control of COVID-19. | People that experienced physical distancing behaviors in the community to prevent/control the COVID-19 pandemic or other similar infectious diseases. | The implementation and planning of risk communication impact the behavior of individuals and the community. Some resources can increase adherence to community measures in general, including physical distance, such as: i. Providing clear, accurate, timely and up-to-date information on aspects of physical distance (benefits and risks) throughout the pandemic period; ii. Use of easy, understandable and consistent language in risk messages, with wide dissemination in different sources; iii. Adaptation of risk messages and community involvement in the preparation and transmission of information. | 27/31 | 8/31 | 27/31 | May  2020 |
| WHO,  2018 | Provide guidance, based on available scientific evidence, on how to effectively practice risk communication in emergency situations. | General population affected by emergencies. | Three interconnected recommendations were identified to achieve the effectiveness of risk communication: i. building trust (risk communication should be clear, easily accessible and comprehensive, widely disseminated across different platforms and channels; ii. Communication of uncertainties (recognizing uncertainties and clearly indicating what is known and unknown at a given time); and iii. participation of community (Identify and include trusted people in the community in the communication process, from planning to dissemination). | Not  reported | Not  reported | Not  reported | End of 2017 |
| Fitzpatric-Lewis et al., 2010 | To identify the effectiveness of communication strategies and factors that impact communication uptake related to environmental health risks | General people from community | Multi-media approach is more effective than a single approach. Printed material that offers a combination of text and diagram is more effective than a single type.  Factors influencing the response to risk communications are: personal risk perception, previous experience with risk, source of information, and trust in these sources. | 2/24 | 0/24 | 4/24 | Nov. 2009 |

^#^ Most of the included studies assessed adherence using non-pooled estimates or p-values without the summary measure or only by qualitative reports of the obtained proportion.

* According to data from World Bank (available at: <https://data.worldbank.org>).

**Table S3.2: Characteristics of included studies about strategy 2 – Health education to the general public**

| **Author, year** | **Objective of study** | **Population of study** | **Main findings ^#^** | **Proportion of studies that included a target population** | **Proportion and identification of studies carried out in low- and middle-income countries*** | **Proportion of problem-focused study** | **Date of the search** |
| --- | --- | --- | --- | --- | --- | --- | --- |
| Li et al., 2020 | To investigate how health providers  should advise families and parents to obtain health education  information on SARS-CoV-2 infection. | General public facing the COVID-19, SARS or MERS pandemic. | The review did not summarize the findings, it was limited to descriptions of the findings of the individual studies included.  The following were highlighted throughout the review: i. Health education improved knowledge, attitude and practice towards COVID-19, increased public awareness and contributed to mitigate negative emotions; and, ii. Health-related information was needed to raise awareness and this was more accurate if it was made from academic, government, or non-profit websites. | 24/24 | 0/24 | 24/24 | March 2020 |
| Cusack et al., 2018 | To identify and evaluate educational interventions designed to improve people’s understanding of key concepts for evaluating claims about the effects of health interventions | Students of both sexes, of any age. | The effect of health education on outcomes related to knowledge and skills development was better in the intervention group than in the control group, measured in the short term (2-6 weeks).  Outcomes related to trust, knowledge perception, attitudes, behavior and satisfaction were not statistically significant. | Not reported clearly | 4/24 | 24/24 | Jan.  2018 |
| Solhi et al., 2017 | To analyze educational interventions used in the area of emerging infectious diseases and the effect of these interventions on preventing and reducing the incidence of these diseases. | Individuals undergoing health education interventions, which emphasized preventing or reducing the incidence of emergency infectious diseases (e.g., Influenza, AIDS, hepatitis B). | Individuals undergoing health education interventions that emphasized preventing or reducing the incidence of emergency infectious diseases (e.g., Influenza, AIDS, hepatitis B).  The review did not summarize the findings, it was limited to describing the findings of the individual studies included. Thus, training in a community setting for similar groups, web-based methods (e-learning or web-based education) and health education combined with approaches to improve health were highlighted as the main interventions that had positive effects in awareness. | 6/16 | 3/16 | 2/16 | Jul.  2017 |
| Nordheim et al., 2016 | To evaluate the effects of educational interventions in schools aimed at enhancing adolescents’ abilities to critically appraise health claims. | Children and adolescents from 11 to 18 years old who attended educational institutions ^35^. | Children and adolescents from 11 to 18 years old who attended educational institutions ^35^.  Health education conducted in schools showed short-term benefits in the effects related to knowledge and skills for critically assessing health-related issues | Not reported clearly | 0/8 | 8/8 | Apr. 2016 |

^#^ Most of the included studies assessed adherence using non-pooled estimates or p-values without the summary measure or only by qualitative reports of the obtained proportion.

* According to data from World Bank (available at: <https://data.worldbank.org>)

**Table S3.3: Characteristics of included studies about strategy 3 - Financial support and access to essential supplies and services.**

| **Author, year** | **Objective of study** | **Population of study** | **Main findings ^#^** | **Proportion of studies that included a target population** | **Proportion of studies carried out in low- and middle-income countries*** | **Proportion of problem-focused study** | **Date of the search** |
| --- | --- | --- | --- | --- | --- | --- | --- |
| Ryan et al., 2020 | To identify relevant, viable and effective approaches to promote acceptability, understanding and adherence to physical distancing measures for the prevention and control of COVID-19. | General population that experienced physical distancing behaviors in the community to prevent/control the COVID-19 pandemic or other similar infectious disease | The guarantee of financial support and access to essential supplies and services, such as food, medication and medical services, provides conditions for the population to adhere to physical distance and, in turn, increases adherence. Support and access to essential supplies and services must be tailored to reach all population groups. | 27/31 | 8/31 | 27/31 | May 2020 |
| Webster et al., 2020 | To identify factors associated with adherence to quarantine during infectious disease outbreaks. | Individuals who were placed in quarantined outside the hospital environment for at least 24 hours | Quarantine adherence ranged from 0 to 92.8% and the main factors associated with increased adherence to quarantine were: i. knowledge about the disease and the quarantine protocol; ii. Perception of benefits; iii. risk perception; iv. Maintenance of practical aspects (work, family income, essential supplies and medical care); v. sociocultural factors (social norms, social pressure to adhere, cultural values and law); iv. individual characteristics; | 5/14 | The countries of all included studies were not reported. | 5/14 | Jan.  20 |

^#^ Most of the included studies assessed adherence using non-pooled estimates or p-values without the summary measure or only by qualitative reports of the obtained proportion.

* According to data from World Bank (available at: <https://data.worldbank.org>)
