# Supplementary material for: Improving the adherence to COVID-19 preventive measures in the community: Evidence brief for policy
Source: Front Public Health. 2022 Aug 1;10:894958. doi: 10.3389/fpubh.2022.894958 (PMC9376604; doi:10.3389/fpubh.2022.894958)
Supplement: Supplementary file 5 [file Table_4.DOCX]

**SUPPLEMENTARY TABLE S4 – ASSESSMENT OF INCLUDED GUIDELINE**

**Table S4.1: Result of the assessment of the World Health Organization policy guideline in accordance with AGREE II.**

| **AGREE II domains** | **Total items score for each domain** | | | **Final score for each domain**  **% (100)** |
| --- | --- | --- | --- | --- |
|  | **Appraiser**  **1** | **Appraiser**  **2** | **Appraiser**  **3** |  |
| **Domain 1 (items 1-3):**  **Scope and purpose** | 21 | 21 | 21 | 100 |
| **Domain 2 (items 4-6):**  **Stakeholders involvement** | 18 | 21 | 19 | 90,7 |
| **Domain 3 (items 7-14):**  **Rigour of development** | 41 | 55 | 49 | 84,1 |
| **Domain 4 (items 15-17):**  **Clariy e presentation** | 21 | 21 | 20 | 98,2 |
| **Domain 5 (items 18-21): Applicability** | 26 | 28 | 25 | 93,1 |
| **Domain 6 (items 22-23):**  **Editorial Independence** | 12 | 14 | 14 | 59,7 |

**Table S4.2: Evaluation of the World Health Organization guideline according to the guideline quality global ranking item and the guideline use recommendation item.**

| **Items** | **Appraiser**  **1** | **Appraiser**  **2** | **Appraiser**  **3** | **Mean** |
| --- | --- | --- | --- | --- |
| **Overall quality of this guideline** | 6/7 | 7/7 | 6/7 | 6,3 |
| **Guideline recommended for use?** | yes | Yes | yes | yes |
